# Foreword to the focus issue, ’nanoarchitectonics reloaded: method for everything in materials science’

**DOI:** 10.1080/14686996.2025.2607212

**Published:** 2026-01-23

**Authors:** Katsuhiko Ariga

**Affiliations:** a Research Center for Materials Nanoarchitectonics, National Institute for Materials Science (NIMS), Tsukuba, Japan; b Graduate School of Frontier Sciences, The University of Tokyo, Kashiwa, Japan

This foreword provides an introduction to the focus issue, ‘Nanoarchitectonics Reloaded: Method for Everything in Materials Science’. To this end, it will explore the significance of nanoarchitectonics in the history of materials science and consider whether it could become a universal method in this field as a method for everything in materials science [1,2].

The search for and development of new substances and materials presents humanity with a constant challenge. Human society’s development is deeply dependent on the evolution of material civilization. Useful materials and the convenient tools created from them can greatly improve the quality of life. The development process began with extracting materials from nature, processing them, and putting them to use. Based on experience, humanity has modified substances through various processes to develop new materials, such as alloys. In the 20th century, various academic fields related to materials science developed rapidly, enabling the rational development of functional materials. These materials meet a wide range of needs, including those in the fields of energy [3,4], the environment [5,6], and biomedicine [7,8].

Thanks to advances in the scientific field, we now understand that substances and materials can be created by materials science. However, the situation is not so simple. It has become clear that the nanoscale structure and effects of even the same material can significantly alter its function. In other words, achieving higher functionality requires more than simply synthesizing a material; precise control of its structure and internal organization is also necessary. To do so requires a better understanding of phenomena at the atomic, molecular and nanoscale levels. This has become a new challenge. Nanotechnology, which has developed rapidly since the second half of the 20th century, has greatly promoted this trend. High-resolution microscopes and measurement techniques enable us to observe atoms and molecules directly [9,10], and to investigate and manipulate the detailed properties of nanoscale phenomena [11,12].

Nanotechnology has revealed the science of the nanoscale. The next challenge is to apply this knowledge to the construction of functional materials. The concept of nanoarchitectonics has been proposed to accomplish this task [13]. This concept involves using atoms, molecules and nanomaterials as building blocks to construct functional material systems. It also integrates nanotechnology with other fields related to material creation.

Nanoarchitectonics is often a multi-step process that can easily form asymmetric, hierarchical structures. Compared to self-assembly through a simple equilibrium process, nanoarchitectonics offers the potential for a wider variety of structural constructions. Furthermore, the underlying nano-level interactions include uncertainties such as thermal fluctuations, probabilistic distributions and quantum effects [14]. Rather than accumulating interactions, it is a construction technology that harmonizes the whole. The formation of hierarchical structures and the coexistence of elements such as thermal fluctuations are similar to the advanced structural organization found in living organisms [15].

Therefore, its applications are wide-ranging. The papers collected in this special issue, entitled Nanoarchitectonics Reloaded, cover a variety of topics, as outlined below.　The indexed research topics are diverse with including various materials from different fields. For inorganic and related fields, ‘supra-ceramics as a frontier of inorganic materials’ [16], ‘Ce-based solid-phase catalysts’ [17], ‘millimeter-scale ZIF-8 single crystals’ [18], ‘lightweight acoustic hyperbolic diaphragms with graphene’ [19], and ‘polymer-coating on carbon nanotubes’ [20] are included. Research activities based on organic compounds, polymeric materials, and the other related ones are also subjected, including ‘π-extended anion-responsive organoplatinum complexes’ [21], ‘thermo-responsive polymer gels composed of star-shaped block copolymers’ [22], ‘physicochemical properties of pyrrolidinium-based electrolytes’ [23], ‘hydrogen peroxide sensors based on ordered carbonaceous frameworks from iron porphyrin’ [24], and ‘RGB circularly polarized luminescence from solid microspheres’ [25]. Even targets of this issue are expanded to biomaterials-based research such as ‘cello-oligosaccharides for fabricating functional nonwoven fabrics’ [26], ‘biofabrication of engineered blood vessels’ [27], ‘decellularized extracellular matrix based materials’ [28], ‘enzymatic control of intermolecular interactions in cellular environment’ [29], and ‘artificial viral capsid budding outside-to-inside and inside-to-outside giant vesicles’ [30].

All matter is essentially made up of atoms and molecules. Therefore, nanoarchitectonics, the construction of matter from atoms and molecules, could be a universal method for creating all matter. This would correspond to the Theory of Everything in physics. In other words, it could be called the Method for Everything in materials science. This methodology could be considered the ultimate challenge in materials science. It would not be a bad thing for us materials scientists, to aim for the completion of materials nanoarchitectonics as a universal methodology for everything in functional materials as our ultimate challenge for the future.
